# Supplementary material for: PUFFIN: protein unit discovery with functional supervision
Source: Bioinformatics. 2026 Jul 7;42(Suppl 1):btag265. doi: 10.1093/bioinformatics/btag265 (PMC13340179; doi:10.1093/bioinformatics/btag265)
Supplement: btag265_Supplementary_Data [file btag265_supplementary_data.pdf]

SUPPLEMENTARY MATERIALS FOR: PUFFIN: PROTEIN UNIT DISCOVERY WITH FUNCTIONAL SUPERVISION

1 Unit Cluster Construction

After end-to-end training, we extract embeddings for all active units in the training set. Let $\{\mathbf{e}_m\}_{m=1}^{M}$, $\mathbf{e}_m \in \mathbb{R}^H$, denote the resulting set of unit embeddings across proteins.

Unit embeddings are ℓ_2-normalized and transformed using a debiasing procedure fitted on the training set:

$$\mathbf{e}'_m = \mathrm{norm}\left(\mathbf{e}_m - \boldsymbol{\mu} - \sum_{r=1}^{R} \langle \mathbf{e}_m - \boldsymbol{\mu}, \mathbf{p}_r \rangle \, \mathbf{p}_r \right), \tag{1}$$

where $\boldsymbol{\mu}$ is the mean embedding and $\{\mathbf{p}_r\}_{r=1}^{R}$ are the top principal components of centered training embeddings. The same transform is applied to validation and test units.

We learn P global clusters by clustering the transformed training embeddings $\{\mathbf{e}'_m\}$ using spherical k-means, yielding unit-norm centroids $\{\mathbf{c}_p\}_{p=1}^{P}$. Each unit embedding is assigned to its nearest prototype using cosine similarity.

Cluster learning is performed using spherical k-means on the unit embeddings and used $n_{\text{init}} = 5$ random initializations and 40 update iterations. We explore different number of clusters $P \in \{128, 256, 512, 1024, 2048, 4096\}$. The optimal value of P is selected using a joint scoring criterion that balances prototype coverage and functional specificity, detailed in Section .

2 Evaluation

The evaluation of PUFFIN involves several aspects: i) characterization of units, ii) functional analysis of units and their corresponding embeddings, iii) assessment of its function prediction performance. Additionally, unit clusters are characterised by their usage and functional enrichment.

2.1 Protein unit evaluation

We assess protein units identified by PUFFIN along two complementary axes: (i) their granularity, sequence contiguity, and structural compactness and separation and (ii) the functional coherence of their learned embeddings with respect to GO annotations.

Characterization metrics. Let a protein consist of N residues indexed by $i = 1, \dots, N$, with hard unit assignments

$$a_i = \arg\max_{m \in \{1,\dots,M\}} s_{im},$$

and let M denote the set of active unit defined previously. For a given protein, the residue set of unit m is

$$\mathcal{R}_m = \{\, i \in \{1, \dots, N\} \mid a_i = m \,\}, \qquad m \in M.$$

The size of unit m is defined as

$$n_{\text{res}}(m) = |\mathcal{R}_m|.$$

Sequential coherence. To quantify fragmentation in sequence order, we define the number of contiguous runs of unit m as

$$r_m = 1 + \sum_{i=2}^{N} \mathbb{I}[a_i = m \;\wedge\; a_{i-1} \neq m] \,,$$

where $\mathbb{I}[\cdot]$ denotes the indicator function. We report the normalized fragmentation ratio

$$\mathrm{frag}(m) = \frac{r_m}{n_{\mathrm{res}}(m)},$$

with lower values indicating higher sequence contiguity.

Structural compactness and connectivity. Let $\mathbf{x}_i \in \mathbb{R}^3$ denote the Cα coordinate of residue i, and let $\mathcal{C}$ denote the set of residue–residue contacts.

For each unit m, the mean intra-unit Cα distance is computed as

$$d_{\mathrm{intra}}(m) = \frac{1}{|\mathcal{R}_m|(|\mathcal{R}_m| - 1)} \sum_{\substack{i,j \in \mathcal{R}_m \\ i \neq j}} \|\mathbf{x}_i - \mathbf{x}_j\|_2.$$

We further define the cut ratio of unit m as the fraction of contacts crossing the unit boundary:

$$\mathrm{cut}(m) = \frac{\big|\{(i,j) \in \mathcal{C} \mid a_i = m,\ a_j \neq m\}\big|}{\big|\{(i,j) \in \mathcal{C} \mid a_i = m\}\big|}.$$

Functional coherence evaluation We evaluate whether learned unit embeddings capture functionally meaningful patterns using a k-nearest neighbor (kNN) analysis in embedding space. Each active unit m from protein p is associated with an embedding $\mathbf{z}_{p,m} \in \mathbb{R}^d$. For each unit embedding, we retrieve its neighborhood $\mathcal{N}_m$ consisting of the k nearest unit embeddings under cosine similarity. Each neighbor $j \in \mathcal{N}_m$ originates from a protein denoted $p(j)$.

Let $\mathcal{G}_p$ denote the set of Gene Ontology (GO) terms annotating protein p. We quantify functional coherence of unit neighborhoods using the following metrics.

Neighborhood overlap. Neighborhood overlap measures the fraction of unit proteins sharing at least one GO term with the query protein:

$$\mathrm{overlap}(m) = \frac{1}{|\mathcal{N}_m|} \sum_{j \in \mathcal{N}_m} \mathbb{I}\big(\mathcal{G}_{p(j)} \cap \mathcal{G}_p \neq \emptyset\big).$$

Higher values indicate neighborhoods enriched for functionally related proteins.

GO term enrichment. To assess finer-grained functional specificity, we test each GO term $g \in \mathcal{G}_p$ for over-representation among neighbor proteins relative to the background protein set of the evaluation split. For each term, enrichment significance is assessed using a one-sided Fisher exact test. For each unit m, we report the most significant enrichment scores among the query protein's terms:

$$p^*(m) = \min_{g \in \mathcal{G}_p} p_{\mathrm{Fisher}}(g).$$

Cluster evaluation We further evaluate learned unit clusters. Each unit is assigned to its nearest cluster embedding, and cluster usage is characterized by the distribution of assigned units across proteins.

We perform functional enrichment analysis at the cluster level by testing GO term over-representation among proteins contributing units to each prototype, using a Fisher exact test analogous to the unit neighborhood analysis.

Finally, we assess the correspondence between discovered units and known functional annotations by comparing prototype-associated units to InterPro regions. InterPro annotations are mapped to GO terms via InterPro2GO. We rank clusters for each annotation based on GO term enrichment, and report standard retrieval metrics including mean reciprocal rank (MRR), Hit Rate@K, precision, and recall.

2.2 Function prediction metrics

We evaluate function prediction performance using the standard metrics: the maximum F-measure (F_{max}), and the area under the precision-recall curve (AUPRC).

The maximum F-measure (F_{max}) is calculated as the maximum harmonic mean of precision and recall across different confidence thresholds t:

$$F_{\max} = \max_t \left\{ \frac{2 \cdot pr(t) \cdot rc(t)}{pr(t) + rc(t)} \right\} \tag{2}$$

where precision $pr(t)$ and recall $rc(t)$ at threshold t are defined as:

$$pr(t) = \frac{1}{m(t)} \sum_{i=1}^{m(t)} \frac{|P_i(t) \cap T_i|}{|P_i(t)|} \tag{3}$$

$$rc(t) = \frac{1}{n} \sum_{i=1}^{n} \frac{|P_i(t) \cap T_i|}{|T_i|} \tag{4}$$

where $P_i(t)$ represents the set of predicted GO terms with confidence score $\geq t$ for protein i, T_i is the set of true GO terms for protein i, n is the number of proteins with at least one GO term annotation, and $m(t)$ is the number of proteins with at least one prediction at threshold t.

For function-centric evaluation, we employ a macro-averaging approach across all GO terms, calculating the Area Under the Precision-Recall Curve (AUPRC) for each term separately:

$$AUPRC_j = \sum_{k=1}^{n-1} (r_{j,k+1} - r_{j,k}) \cdot \frac{p_{j,k+1} + p_{j,k}}{2} \tag{5}$$

where $p_{j,k}$ and $r_{j,k}$ are the precision and recall values at the k-th threshold for GO term j. The macro-averaged metrics are then computed as:

$$M - AUPRC = \frac{1}{|J|} \sum_{j=1}^{|J|} AUPRC_j \tag{6}$$

where $|J|$ is the total number of GO terms.

2.3 Cluster evaluation

We evaluate cluster quality by examining the enriched GO terms associated with each cluster after clustering and then computing coverage, functional enrichment, and cluster diversity for them.

Let K denote the number of clusters, $\mathcal{C} = \{C_1, \ldots, C_K\}$ the set of all units mapping to each cluster, $\mathcal{G}_{all}$ the set of all GO terms observed in the training set, and the function $\mathcal{S}(k)$ returning the set of all enriched GO terms for cluster k.

2.3.1 GO Coverage

GO coverage measures the fraction of enriched GO terms that are represented by at least one cluster:

$$GC(K) = \frac{\left|\bigcup_{i=1}^{K} \mathcal{S}(C_i)\right|}{|\mathcal{G}_{all}|}, \tag{7}$$

where $\mathcal{G}(C_i)$ denotes the set of enriched GO terms associated with the cluster C_i. Higher values indicate broader functional coverage.

2.3.2 GO per cluster

This metric computes the mean number of enriched GO terms per cluster:

$$GpC(K) = \frac{1}{K} \sum_{i=1}^{K} |\mathcal{S}(C_i)|. \tag{8}$$

Lower values indicate more functionally specific clusters.

2.3.3 Clusters per GO

Clusters per GO measures the average number of clusters for which a GO term is enriched:

$$CpG(K) = \frac{1}{|\mathcal{G}_{all}|} \sum_{g \in \mathcal{G}_{all}} |\{C_i \mid g \in \mathcal{S}(C_i)\}|. \tag{9}$$

Lower values reflect reduced functional fragmentation across clusters.

2.3.4 Enriched Cluster Fraction (N/K)

The fraction of enriched clusters is then defined as:

$$ECF(K) = \frac{|\{C_i \mid |\mathcal{S}(C_i)| > 0\}|}{K} \tag{10}$$

where N is the number of clusters containing at least one enriched GO term. Values close to 1 indicate that most clusters are functionally meaningful.

2.3.5 Joint Score

The joint score combines coverage, specificity, and enrichment consistency into a single scalar objective:

$$\text{Joint}(K) = \frac{GC(K)\ \log(1 + ECF(K))}{\log(1 + GpC(K))^2\ \log(1 + CpG(K))}, \tag{11}$$

where $GpC(K)$ is more hardly penalised as functionally specific clusters are more important than redundancy.

3 Hyperparameter selection

3.1 Unit Size

We explore models with varying maximum numbers of units per protein, denoted by $M \in \{16, 32, 64\}$. We evaluate how unit properties change as M increases, focusing on diversity, fragmentation, structural coherence, compactness, and size-related characteristics.

Figure S1 clarifies the role of M, showing the effective number of units used when segmenting test proteins. Although M defines the maximum number of units that can be allocated, the model partitions a protein into fewer units and adapts the segmentation to each protein in practice.

Figure S2 summarizes the results for MinCut-based segmentation and PUFFIN . Both methods show similar qualitative trends across M. However, PUFFIN consistently favors functionally coherent segmentation, often at the expense of stricter structural constraints.

At lower values of M, both methods achieve high segment diversity, while a decrease is observed at $M = 64$, more pronounced for PUFFIN . This suggests increasing specialization of units as the number of units per protein grows.

Fragmentation behavior is comparable between methods at $M = 16$ and $M = 32$. At $M = 64$, PUFFIN exhibits lower fragmentation than MinCut, indicating more contiguous units along the sequence at higher granularity.

Structural coherence improves for both methods as M increases, with PUFFIN showing a larger improvement at $M = 64$. In contrast, structural compactness exhibits method-specific behavior, where PUFFIN forms larger and less compact segments at higher K, while MinCut maintains more compact units.

Consistent with these trends, PUFFIN produces larger units with longer sequence spans at $M = 64$ compared to MinCut, reflecting a preference for fewer, longer units rather than uniformly small fragments.

3.2 Cluster Size

We select the cluster size by jointly considering coverage, functional enrichment, and cluster diversity using Equation 11. Figure S3 shows how these criteria change as the number of prototypes increases.

We identify the K value, 1024, that balances coverage, specificity, and robustness of functional annotation.

GO coverage stays roughly constant as the number of clusters increases, while enrichment-related measures, especially the fraction of enriched clusters, degrade at large cluster sizes. At the same time, larger cluster sets reduce diversity within clusters but increase cluster redundancy.

The joint score peaks at an intermediate cluster size, reflecting a trade-off between broad coverage and meaningful functional enrichment. Based on this analysis, we select the cluster size corresponding to the maximum joint score.

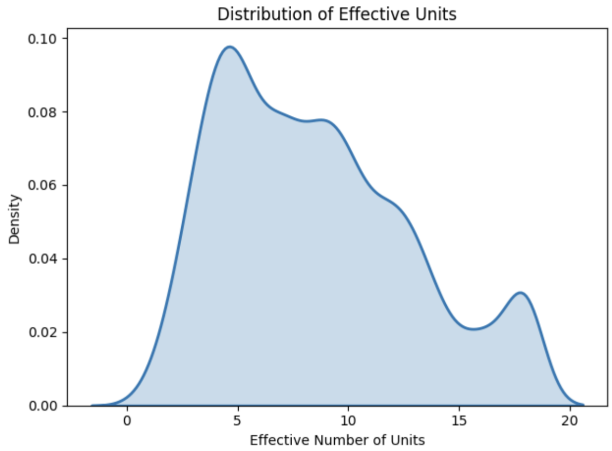

Figure S1: Effective number of units used in segmenting test proteins.

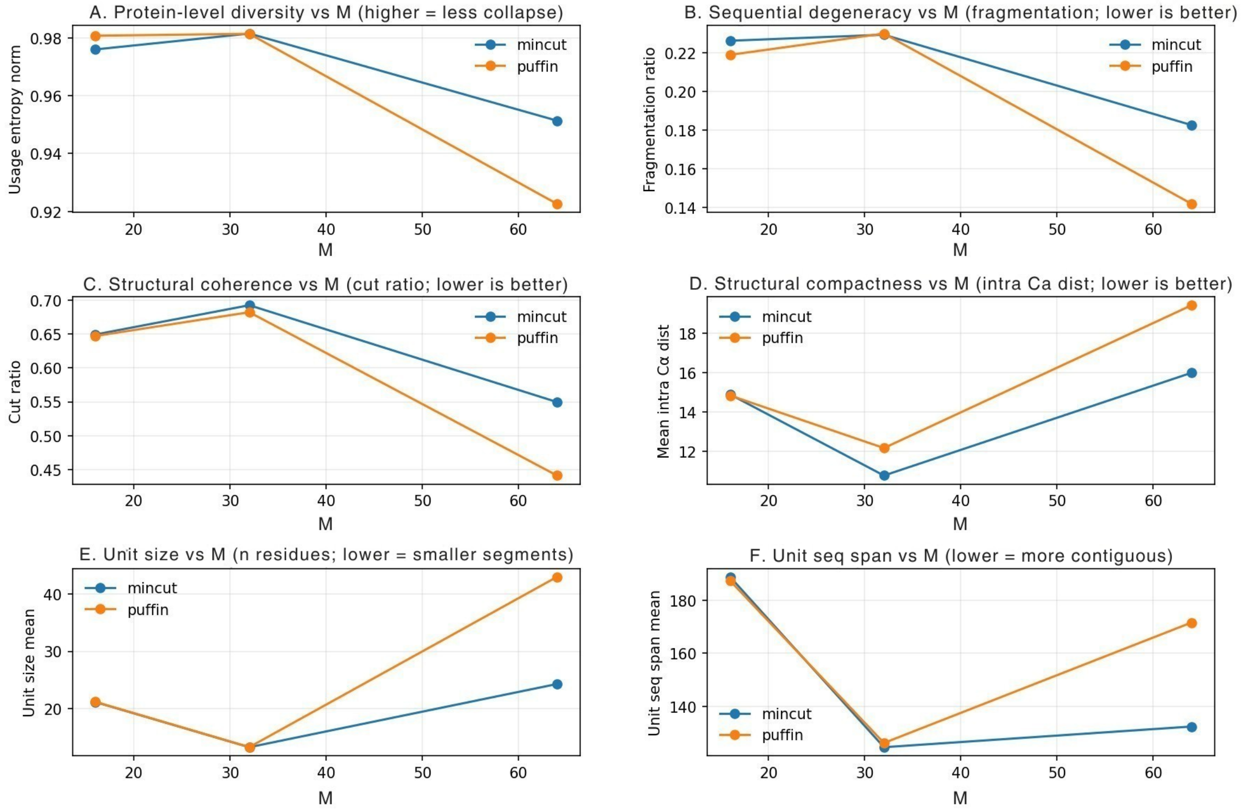

Figure S2: Unit characterization.

3.3 InterPro retrieval performance

Comprehensive retrieval metrics are reported to enable comparison with InterPro annotations across the various annotation categories (Table S1). Since a single InterPro annotation may map to multiple GO terms, the evaluation is performed in a multilabel setting, with the InterPro2GO mapping as the reference label set.

Hit@k measures whether at least one reference GO term appears among the top-k enriched GO terms for the matched prototype. Precision@k is defined as the fraction of the top-k predicted GO terms that are present in the reference

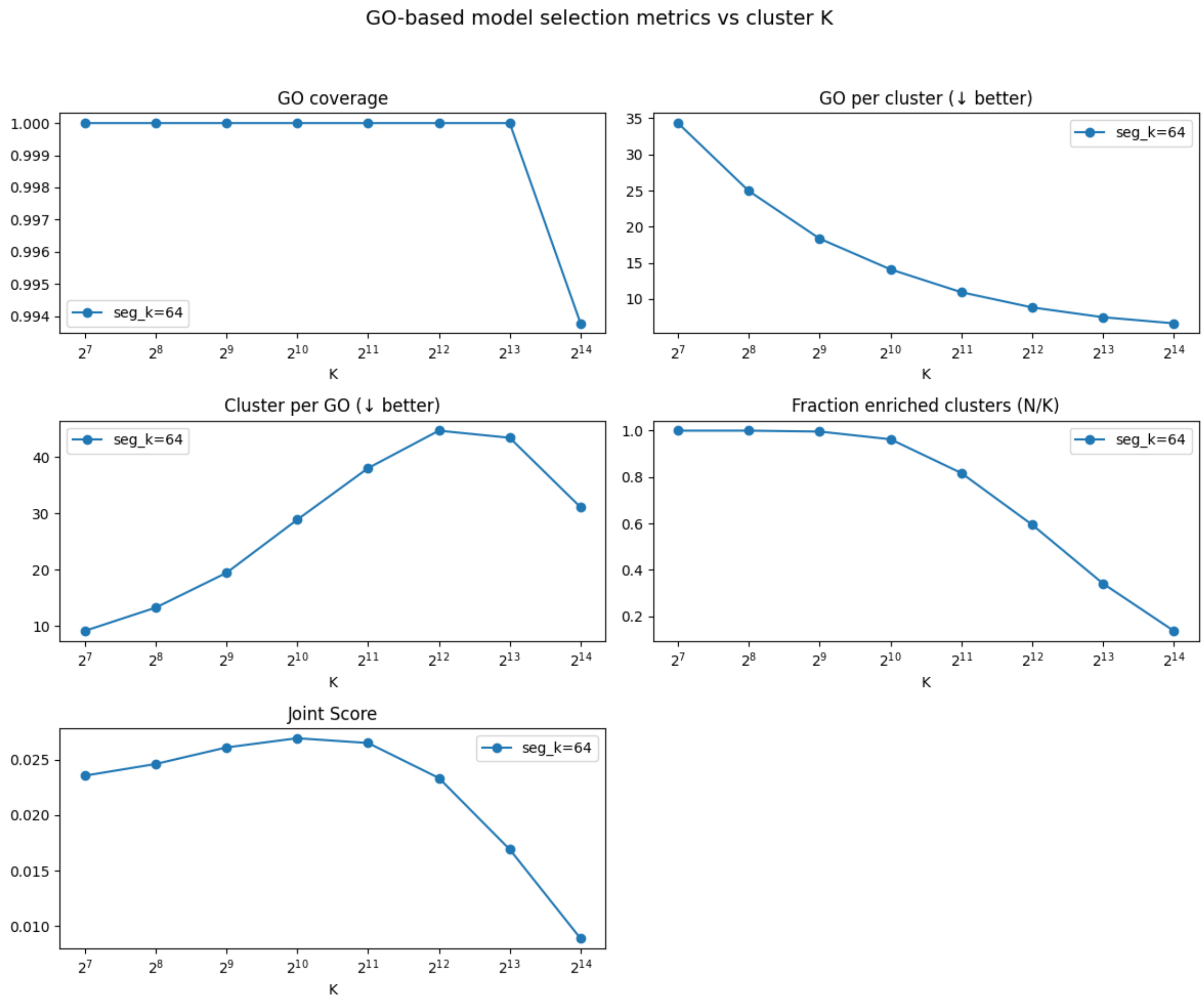

Figure S3: Cluster k was selected by observing GO enrichment metrics.

set, while Recall@k measures the fraction of reference GO terms that are recovered within the top-k predictions. In addition, mean reciprocal rank (MRR) is reported to capture the rank of the first correctly recovered GO term.

3.4 Case Study on Short-chain Dehydrogenase/Reductase (SDR) Protein Family

To assess whether PUFFIN-derived unit clusters are conserved among protein families, we performed an analysis on Short-chain Dehydrogenase/Reductase (SDR) protein family (IPR002347). The SDR family is a large family of enzymes most of which function as oxidoreductases. These proteins display high sequence diversity and perform a vast range of metabolic roles, making them an ideal family for an analysis of the PUFFIN-derived units. Our analysis included all 25 SDR-labeled proteins in the test set, without any exclusions.

3.4.1 Unit Cluster Visualisation on Protein Sequence

To visualize high-dimensional unit representations, we implemented a deterministic mapping of unit cluster centroids into the RGB color space. Centroid embeddings were projected onto their first three principal components using Principal Component Analysis (PCA) to capture primary structural variance. These components were then globally min-max normalized to a $[0, 1]$ range.

$$C_{norm} = \frac{C_{pca} - \min(C_{pca})}{\max(C_{pca}) - \min(C_{pca}) + \epsilon} \tag{12}$$

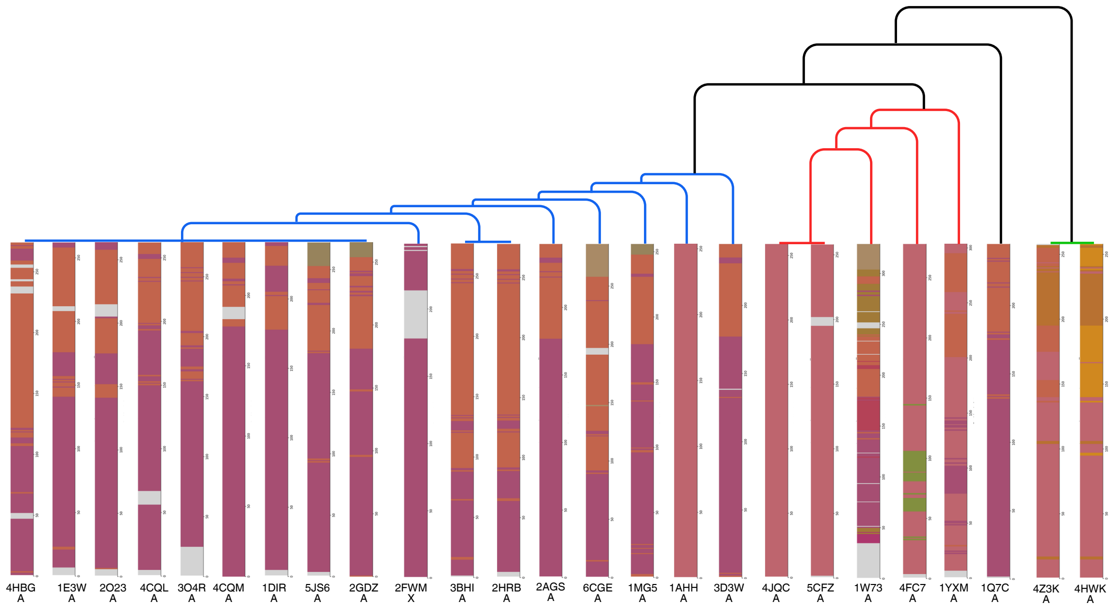

Figure S4: **Hierarchical Clustering and Sequence Segmentation Maps of the SDR Protein Family.** The dendrogram (top) illustrates the relationships between various SDR proteins based on their **InterPro accession-based clusters derived with Jaccard scores**. The branching colors (blue, red, and green) indicate distinct sub-groups identified. Below each leaf of the dendrogram is a **protein sequence unit cluster visualisation** for a specific protein. These vertical bars represent the residue index, with different colors representing their corresponding PUFFIN-based unit clusters.

The resulting values were mapped to red, green, and blue channels respectively, and converted to hexadecimal codes. This ensures that unit clusters with similar features are represented by perceptually similar colors across all proteins.

3.4.2 Comparison with InterPro Annotations

To compare the unit clusters assigned by PUFFIN with annotations from InterPro, we conducted a *hierarchical clustering* analysis of the 25 SDR proteins based on their associated sets of InterPro annotations, using the Jaccard similarity as the similarity measure. A distance threshold of 0.5 was applied to the resulting dendrogram, which yielded four distinct clusters, one of which contained a single protein and was excluded from further consideration in our analysis. We then visualized the dendrogram generated by the *hierarchical clustering* procedure alongside the protein sequence unit cluster visualization for each protein, which employs the PCA-based color assignment approach described above.

The assigned colors are largely conserved across all proteins, indicating that the representations of units within a given protein family are generally similar. Across these proteins, 11 distinct unit clusters are identified, with clusters 917, 670, and 281 being the most prevalent. Clusters 917 and 670 share the same three most frequent GO annotations: oxidoreductase activity, acting on the CH-OH group of donors (GO:0016614); oxidoreductase activity, acting on the CH-OH group of donors, NAD or NADP as acceptor (GO:0016616); and fatty acid synthase activity (GO:0004312). By contrast, the top three GO annotations for cluster 281 are fatty acid synthase activity (GO:0004312); oxidoreductase activity, acting on the CH-CH group of donors, NAD or NADP as acceptor (GO:0016628); and oxidoreductase activity, acting on the CH-CH group of donors (GO:0016627). Another cluster of interest is cluster 71, with GO terms hydro-lyase activity (GO:0016836), oxidoreductase activity, acting on the CH-OH group of donors, NAD or NADP as acceptor (GO:0016616), and O-acyltransferase activity (GO:0008374). The remaining clusters occur less frequently and consequently occupy a smaller fraction of the protein sequences.

There are 17 proteins assigned to the blue group. With the exception of *1AHH*, which appears to represent a misassigned unit cluster, all of these proteins share similar unit cluster compositions. Looking at the GO annotations of these proteins, we see that this cluster predominantly contains SDRs that catalyze reactions involving a $C = O$ bond, consistent with the GO terms of the dominant clusters 917 and 670 utilized in this group. Although these two clusters share similar GO terms, their separation, with cluster 917 localized to the N-terminal region and cluster 670 to the C-terminal region, suggests a structural and functional distinction within the protein. This distinction aligns with the literature, which describes the N-terminal region as the cofactor-binding domain and the C-terminal region as the substrate-binding

domain Kallberg et al. [2002]. It is also noteworthy that, based on their GO annotations, *1DIR* acts on $CH\text{-}NH$ groups and *2FWM* acts on $CH\text{-}CH$ groups. This observation suggests that the use of InterPro-based annotations does not always yield functionally homogeneous or strictly distinct protein clusters, as these two proteins were grouped with enzymes acting on $CH\text{-}OH$.

In the red group, the proteins with PDB identifiers *4JQC, 5CFZ, 1W73, 4FC7*, and *1YXM* are observed. According to the GO annotations of these proteins, these correspond to SDRs that catalyze reactions involving a $C = C$ double bond. All but one of these proteins contain Cluster 281, which is associated with oxidoreductase activity acting on CH–CH groups. Consequently, for this group, the functional assignments generated by our method are consistent with the corresponding InterPro annotations.

The final green group corresponds to the human protein sepiapterin reductase (SPR). The two PDB chains shown represent the same protein bound to different inhibitors. Both structures contain Cluster 71; however, in this particular case, the associated GO terms do not accurately reflect the protein's function. Furthermore, the differences in their unit cluster compositions, for instance, the presence of an additional cluster in *4HWK* relative to *4Z3K*, further illustrate the sensitivity of PUFFIN to structural variations induced by ligand binding.

Overall, our analysis of the SDR family demonstrates that PUFFIN can detect functionally relevant signals, such as detailed characteristics of oxidoreductase activity, and assign functional units accordingly. The presence of some incorrect unit cluster compositions and GO term assignments indicates that there is still room for improvement; however, this may be inherently challenging due to the noisy and potentially inconsistent functional annotations in InterPro.

References

Y. Kallberg, U. Oppermann, H. Jörnvall, and B. Persson. Short-chain dehydrogenases/reductases (sdrs). *European Journal of Biochemistry*, 269(18):4409–4417, 2002. doi: https://doi.org/10.1046/j.1432-1033.2002.03130.x. URL `https://febs.onlinelibrary.wiley.com/doi/abs/10.1046/j.1432-1033.2002.03130.x`.

Table S1: Comprehensive InterPro retrieval performance metrics across all categories. Best results per category and metric are in bold. Values in parentheses next to the InterPro type indicate the number of proteins evaluated. MRR: Mean Reciprocal Rank, H@k: Hit at k, P@k: Precision at k and R@k: Recall at k.

**InterPro Type**	**Model**	**H@1**	**H@3**	**H@5**	**P@1**	**P@3**	**P@5**	**R@3**	**R@5**	**R@10**
active_site (159)	ESM-$k$means	0.3931	0.5063	0.5063	0.3931	0.1688	0.1013	0.5063	0.5063	0.5189
	MinCut	0.1509	0.2390	0.2579	0.1509	0.0797	0.0516	0.2358	0.2547	0.3113
	**PUFFIN**	**0.5723**	**0.6226**	**0.6384**	**0.5723**	**0.2138**	**0.1314**	**0.6226**	**0.6384**	**0.6887**
binding_site (118)	ESM-$k$means	0.0847	0.1441	**0.6271**	0.0847	0.0508	**0.1271**	0.1059	**0.5890**	**0.6419**
	MinCut	0.0339	0.0466	0.2246	0.0339	0.0155	0.0449	0.0360	0.2097	0.2535
	**PUFFIN**	**0.1780**	**0.2373**	0.6017	**0.1780**	**0.0819**	0.1220	**0.1822**	0.5424	0.6017
conserved_site (408)	ESM-$k$means	0.1785	0.2574	0.2949	0.1785	0.1070	0.0776	0.1935	0.2326	0.3015
	MinCut	0.0188	0.0654	0.0923	0.0188	0.0218	0.0185	0.0487	0.0675	0.1023
	**PUFFIN**	**0.3297**	**0.4105**	**0.4939**	**0.3297**	**0.1679**	**0.1262**	**0.3321**	**0.4007**	**0.4412**
domain (1281)	ESM-$k$means	0.2284	0.2856	0.3178	0.2284	0.0970	0.0682	0.2351	0.2701	0.3280
	MinCut	0.0476	0.1002	0.1272	0.0476	0.0341	0.0268	0.0836	0.1080	0.1591
	**PUFFIN**	**0.3094**	**0.3836**	**0.4224**	**0.3094**	**0.1326**	**0.0951**	**0.3354**	**0.3834**	**0.4201**
family (1807)	ESM-$k$means	0.1361	0.2055	0.2422	0.1361	0.0783	0.0586	0.1719	0.2067	0.2468
	MinCut	0.0520	0.0924	0.1084	0.0520	0.0329	0.0245	0.0776	0.0921	0.1234
	**PUFFIN**	**0.2390**	**0.3080**	**0.3635**	**0.2390**	**0.1152**	**0.0855**	**0.2538**	**0.3070**	**0.3551**
homologous_sf (633)	ESM-$k$means	0.1590	0.1958	0.2101	0.1590	0.0887	0.0639	0.1645	0.1905	0.2135
	MinCut	0.0500	0.0895	0.1014	0.0500	0.0363	0.0259	0.0775	0.0917	0.1185
	**PUFFIN**	**0.2310**	**0.2679**	**0.2995**	**0.2310**	**0.1268**	**0.0919**	**0.2433**	**0.2831**	**0.2953**
repeat (111)	ESM-$k$means	**0.0090**	**0.1059**	**0.1059**	**0.0090**	**0.0353**	**0.0212**	**0.0514**	**0.0514**	**0.0634**
	MinCut	0.0000	0.0045	0.0045	0.0000	0.0015	0.0009	0.0023	0.0023	0.0023
	**PUFFIN**	0.0000	0.0090	0.0315	0.0000	0.0030	0.0063	0.0030	0.0143	0.0315
overall (2494)	ESM-$k$means	0.1649	0.2204	0.2548	0.1649	0.0810	0.0594	0.1875	0.2237	0.2611
	MinCut	0.0519	0.0873	0.1065	0.0519	0.0306	0.0232	0.0756	0.0935	0.1292
	**PUFFIN**	**0.2475**	**0.3137**	**0.3634**	**0.2475**	**0.1154**	**0.0846**	**0.2714**	**0.3215**	**0.4002**
